# Supplementary material for: Genome-wide CRISPR Screens in T Helper Cells Reveal Pervasive Crosstalk between Activation and Differentiation
Source: Cell. 2019 Feb 7;176(4):882–896.e18. doi: 10.1016/j.cell.2018.11.044 (PMC6370901; doi:10.1016/j.cell.2018.11.044)
Supplement: Data S2. Processed Data from All the Steps of the Analysis, Related to Figure 1 [file mmc2.zip › supplemental data/motif analysis/Stat6_SRX021632_homer/homerResults/motif6.similar.html]

motif6

## Information for motif6

A
T
C
G
T
C
A
G
A
G
C
T
T
A
G
C
G
T
C
A
G
A
T
C
T
C
A
G
A
C
G
T
T
A
C
G
T
C
G
A
  
Reverse Opposite:  

A
G
C
T
A
T
G
C
T
G
C
A
A
G
T
C
C
T
A
G
A
C
G
T
A
T
C
G
T
C
G
A
A
G
T
C
T
A
G
C
  

|  |  |
| --- | --- |
| p-value: | 1e-576 |
| log p-value: | -1.328e+03 |
| Information Content per bp: | 1.705 |
| Number of Target Sequences with motif | 2124.0 |
| Percentage of Target Sequences with motif | 8.40% |
| Number of Background Sequences with motif | 533.6 |
| Percentage of Background Sequences with motif | 2.21% |
| Average Position of motif in Targets | 100.8 +/- 28.9bp |
| Average Position of motif in Background | 95.2 +/- 69.3bp |
| Strand Bias (log2 ratio + to - strand density) | 0.0 |
| Multiplicity (# of sites on avg that occur together) | 1.09 |
| Motif File: | file (matrix) reverse opposite |

### Similar de novo motifs found

|  |  |  |  |  |  |  |  |
| --- | --- | --- | --- | --- | --- | --- | --- |
| Rank | Match Score | Redundant Motif | P-value | log P-value | % of Targets | % of Background | Motif file |
| 1 | 0.930 | T C A G A G C T G T A C T G C A A G T C T C A G C A G T A T C G T C G A A G T C A G T C A T C G | 1e-566 | -1305.037374 | 7.80% | 1.95% | motif file (matrix) |
| 2 | 0.976 | C T A G A G C T A G T C C G T A A G T C A C T G A C G T A C T G | 1e-491 | -1131.889826 | 4.17% | 0.62% | motif file (matrix) |
| 3 | 0.634 | C T G A G A C T A T C G C T G A T A G C C A T G A G C T T G A C | 1e-473 | -1090.320605 | 29.47% | 17.51% | motif file (matrix) |
| 4 | 0.817 | T G A C A T G C A G T C C G T A A G T C C T A G A C G T A C T G | 1e-226 | -521.406194 | 5.23% | 1.89% | motif file (matrix) |
